# PRICE: Software for the Targeted Assembly of Components of (Meta) Genomic Sequence Data

**DOI:** 10.1534/g3.113.005967

**Published:** 2013-05-01

**Authors:** J. Graham Ruby, Priya Bellare, Joseph L. DeRisi

**Affiliations:** * Department of Biochemistry and Biophysics, University of California, San Francisco, California 94044; ‡ GW Hooper Foundation Laboratories, University of California, San Francisco, California 94044; † Howard Hughes Medical Institute, Chevy Chase, Maryland 20815

**Keywords:** KSHV, de novo genome assembly, high-throughput DNA sequencing, metagenomics

## Abstract

Low-cost DNA sequencing technologies have expanded the role for direct nucleic acid sequencing in the analysis of genomes, transcriptomes, and the metagenomes of whole ecosystems. Human and machine comprehension of such large datasets can be simplified via synthesis of sequence fragments into long, contiguous blocks of sequence (contigs), but most of the progress in the field of assembly has focused on genomes in isolation rather than metagenomes. Here, we present software for paired-read iterative contig extension (PRICE), a strategy for focused assembly of particular nucleic acid species using complex metagenomic data as input. We describe the assembly strategy implemented by PRICE and provide examples of its application to the sequence of particular genes, transcripts, and virus genomes from complex multicomponent datasets, including an assembly of the BCBL-1 strain of Kaposi’s sarcoma-associated herpesvirus. PRICE is open-source and available for free download (derisilab.ucsf.edu/software/price/ or sourceforge.net/projects/pricedenovo/).

In the past decade, the cost of DNA sequence determination has diminished by orders-of-magnitude due to the maturity of novel technologies (Glenn 2011). Much effort has been applied to the development of computational methods for the *de novo* assembly of genomes using the type of data generated by these technologies: typically, shorter reads and/or higher error frequencies *vs.* traditional Sanger sequencing (Sanger *et al.* 1977; Glenn 2011). The majority of that effort has focused on the assembly of individual whole genomes (Warren *et al.* 2007; Butler *et al.* 2008; Hernandez *et al.* 2008; Zerbino and Birney 2008; Chaisson *et al.* 2009; Simpson *et al.* 2009; Li *et al.* 2010a), whereas *de novo* assembly for metagenomes—the total genome complement of an entire ecosystem or environmental sample—has been less thoroughly explored. Much of the success of *de novo* genome assembly can be attributed to algorithmic optimizations that take advantage of the properties of single-genome datasets. Many of these properties, and therefore their relevant optimizations, are irrelevant to metagenomic datasets, most notably the evenness-of-coverage across the source genome that is used to error-correct source data and identify repetitive elements that could spawn chimeric contigs (Pevzner *et al.* 2001; Butler *et al.* 2008; Chaisson *et al.* 2009; Schröder *et al.* 2009; Kelley *et al.* 2010; Li *et al.* 2010b; Ariyaratne and Sung 2011; Simpson and Durbin 2012). The greater complexity of metagenomic samples renders many current *in silico* assembly techniques less efficient and less accurate. And where algorithmic improvements have been made, they often require special *in vitro* library construction techniques (Hiatt *et al.* 2010; Pignatelli and Moya 2011).

In addition to providing strings of nucleotide identities, many sequencing platforms provide paired-end information. Paired-end reads derive from the two ends of a library amplicon and thus implicitly include information about the distance between and relative orientation of the two sequences in the molecule from which they derive. Given a contig that represents some fragment of a genomic sequence and a large and complex dataset, paired-end information can be and has been used to simplify the extension of that contig by specifying the subset of data relevant to a local assembly and using it to add sequence length to the termini of the contig (Hossain *et al.* 2009; Rausch *et al.* 2009; Li *et al.* 2010a,b; Ariyaratne and Sung 2011; Etter *et al.* 2011). Reduction of the number of input sequences reduces the number of pairwise comparisons that must be made, thereby reducing both the time required for assembly and the probability of spurious assembly of unrelated sequences. Both of these properties facilitate the use of less-stringent alignment requirements than would be necessary with larger datasets, thereby lowering the amount of data required to ensure a successful assembly. Reduced stringency is a boon whether the sequence of interest is a component of a metagenome or simply a particular region (say, a gene of interest) from a single genome. Furthermore, the size of each local assembly job (a “job” defined as a discrete set of sequences for which assembly into contigs will be attempted) can be used to dynamically scale assembly requirements according to the local coverage, thereby allowing each individual genetic component of a metagenomic mixture to be assembled with efficiency and sensitivity tailored to its own level of coverage and agnostic with respect to the total size of the metagenomic dataset.

One practical application of inexpensive DNA sequencing technology has been the rapid discovery and genomic characterization of novel pathogens, particularly viruses, that may contribute to disease in humans or other organisms (Tang and Chiu 2010; Bexfield and Kellam 2011). These pathogens are generally isolated from diseased tissue samples and thus are found as subsets of complex metagenomic data that also includes host sequence and, commonly, nonpathogenic commensal microflora. Viral DNA or RNA typically comprises only a tiny fraction of the total nucleic acid in such samples, and although the small size of many viral genomes results in high genome coverage even given a small number of reads, the methods of shotgun library preparation and peculiar structural qualities of viral nucleic acids can result in highly uneven coverage across the genome, particularly in the case of RNA viruses (Hansen *et al.* 2010). The work described below was motivated by the need for a tool to address the following two peculiarities of RNA-based metagenomic/metatranscriptomic data in the context of viral genome assembly: (1) highly uneven coverage across an entity that (2) comprises only a tiny portion of a massive, complex, largely irrelevant dataset.

We implemented software for a Paired Read-based Iterative Contig Extension strategy (PRICE) as a single package to repeatedly perform all of the generic tasks of a targeted *de novo* assembly strategy. Each “cycle” of assembly includes the mapping of reads to existing contigs; assembly of the paired-ends of those reads, together with the contig itself, to create a larger contig; the construction of scaffolds linking multiple seed contigs that can then be assembled together into a single sequence; avoidance of spurious assemblies that can be created by multicopy (*i.e.*, repetitive) genetic elements; (optionally) evaluation of the output sequences to determine which is relevant to the original target of the assembly; and the collapse of redundant output sequences. A cycle uses those steps to extend the length of existing contigs, and subsequent cycles repeat the process using the output of the prior cycle as input.

Packaging all of these steps into a single piece of software permitted the optimization of each step of the assembly process by taking algorithmic advantage of the context-specific requirements for each step in ways that would be inappropriate for general-case aligners, assemblers, etc. PRICE was implemented in C++ to compile into a single executable requiring only one external non-STL module (openmp: www.openmp.org), making it highly portable. Every step of the assembly process was highly multithreaded, allowing PRICE to take advantage of the ever-increasing availability of multicore central processing units (CPUs). Here, we describe the assembly strategy implemented by PRICE, discuss the relevance of its features to particular assembly challenges, and provide examples of its application to the targeted assemblies of a single transcript from a transcriptome dataset and virus genomes from multi-genome datasets.

## Materials and Methods

### Software design

Details below; see *Results* for an overview of PRICE assembly. The raw data for genome assembly are a set of DNA strings, each of which derives from the chemical sequencing of an amplified DNA molecule. Those “reads” could be presented to PRICE with or without a set of scores indicating the probability that each nucleotide is called correctly using the fastq or fasta file formats, respectively. Each read is a piece of empirical evidence in support of its reported nucleotide identities. When combining two sequences into a contig, PRICE would internally retain the quantity of evidence in the form of a score. For any sequence, two sets of scores would be carried. The first set of scores indicated, for each nucleotide, the number of reads supporting that nucleotide’s stored identity. The second set of scores indicated, for each pair of adjacent nucleotides, the number of reads supporting the linkage of those two nucleotides by a phosphodiester bond. The input reads would each receive a single count for each score type at each position. In the case of fastq input, the nucleotide identity scores would be the probability of the nucleotide having the indicated identity according to the fastq score at that position. Any nucleotide with a redundant International Union of Pure and Applied Chemistry code was considered to have a score of zero.

When two sequences were combined based on a high-quality alignment to yield a contig, the scores of agreeing nucleotides and phosphodiesters were summed at each position. In the case of nucleotide mismatches, the nucleotide with the greater score would be selected, and the nucleotide score for that position of the new contig would be the difference of the two scores. This approach ensured that scores would never fall below zero and that any nucleotide identity with a majority count in the underlying data would always be the final identity selected, independent of the order in which sequences were combined in a pair-wise manner.

The case of disagreeing phosphodiester bonds (gaps in alignments) was less straightforward because of the unequal number of phosphodiester bonds in each sequence for which there was a disagreement. The simplest case was a single block of nucleotides in one sequence paired to gaps in the other, with aligned nucleotides flanking that block. However, placement of gapped blocks adjacent to one another (*i.e.*, using switching between directly from gaps in sequence A to gaps in sequence B with no intervening aligned nucleotides) is legitimate and will occur when alignments are generated with overly punitive mismatch penalties and milder gap penalties. Gaps were therefore considered as blocks, defined as stretches of alignment with no aligned nucleotides. For each block, the average of all disagreeing phosphodiester bonds along each sequence was calculated and compared to determine the sequence with better data support that would be retained in the resulting contig. The ratio of the lower average divided by the higher average was then multiplied to each disagreeing phosphodiester bond score from the winning sequence, thereby proportionally penalizing the discrepancies and maintaining the principle of scores never being less than zero. The nucleotide scores of inserted positions would not change due to the lack of conflicting nucleotides in the alternative sequence. However, the scores of deleted nucleotides would be erased with those positions in the consensus. Therefore, nucleotide scores could be artificially deflated through repeated deletion and reinsertion in the series of pair-wise assembly steps leading to a final consensus sequence.

The replacement of reads with much longer contig sequences had the potential to expand the complexity of individual assembly jobs beyond manageability. In order to avoid this problem, and also to avoid spurious joining of contigs whose linking reads could also have been mapped to other contig loci, as in the cases of a read deriving either from a repetitive element (in the assembly of a single genome) or from a sequence element that is conserved between otherwise distinguishably distinct genomes (in the assembly of metagenomic mixtures), a limit was imposed for the maximum number of contigs that could replace any one read in an assembly job. In cases where a read mapped to a number of contigs in excess of that limit with tying best-possible scores, those mappings would be erased and ignored in further analysis. In cases in which a read or contig was included in multiple distinct assembly jobs, the possibility arose for the evidence scores of nucleotides in that contig to be amplified in the overall assembly. To avoid such an amplification of apparent evidence for a sequence, the scores of all sequences were normalized to the number of assembly jobs in which they were included. If the assembly jobs themselves represented a redundancy in the assembly, then the collapse of the products would return the scores to their original values (also the case if a contig were brought inappropriately into an assembly job by a spuriously mapped read). Conversely, if the contig represented a consensus sequence for a repetitive element within the source (meta)genome, such normalization would appropriately distribute the evidence scores across the multiple loci to which that evidence had been applied, making it easier for locally-anchored read evidence to override the consensus sequence.

Individual assembly jobs were each performed as a hierarchical series of sequence collapses, with each of the following steps executing assembly strategies of increasing computational complexity and sensitivity. First, identical sequences were collapsed, effectively summing the scores for all reads/contigs with the same nucleotide sequence. Second, near-identical sequences were collapsed, defined as sequences of equal length that could be aligned with no gaps or offset. Third, for strand-specific assembly jobs, sequences with a length shorter than some user-specified threshold were collapsed using a de Bruijn graph-based strategy (see below for more details). Fourth, pairs of sequences were collapsed for which one sequence is a complete subset of the other in the context of an ungapped alignment. Fifth, sequences were collapsed based on ungapped alignments for which neither sequence was required to be fully aligned. Sixth, sequences were collapsed based on gapped alignments, again allowing any extent of overlap meeting the minimum overlap requirement of that assembly job. Each of these steps is described in more detail below.

The first and second collapse steps, the collapse of identical and near-identical sequences, were designed to reduce the complexity of the input sequence pool as efficiently as possible. The underlying assumption of these steps was that the collapsed sequences were redundant pieces of data, and that observed polymorphism should be treated as decreased confidence in the observed nucleotide identities. These assumptions limited the scope of sequence comparisons to ungapped, end-to-end alignments performed only between equal-length sequences. Searches for identical sequences were the easiest to perform, so those sequences were the first to be collapsed. Near-identical matches were then sought by dividing each sequence into a series of nonoverlapping windows and performing comparisons between those pairs of sequences with perfect matches between one or more of their windows.

The third collapse step used a de Bruijn graph-based strategy to collapse short sequences. The confinement of this strategy to short (*i.e.*, read-length) sequences prevented the erasure of long-range structural information contained in large contigs (or a limited number of very long reads). For long sequences, k-mers would not necessarily be unique due to repetitive elements. However, the majority of sequences serving as input for any assembly job were short and derived from a small, local region of the source genome from which repeated k-mers were unlikely to cause global-scale assembly errors. Thus, the highly efficient de Bruijn method could be applied safely to the short sequences of localized assembly jobs.

A plethora of variants of the de Bruijn assembly strategy provide the core algorithms for many of the existing *de novo* genome assemblers (Pevzner *et al.* 2001; Butler *et al.* 2008; Zerbino and Birney 2008; Simpson *et al.* 2009; Li *et al.* 2010b). Much of the complexity of those algorithms derives from the double-stranded nature of DNA, which forces each k-mer to be simultaneously considered as two different k-mers: the observed k-mer and its reverse complement. The biological meaning of the de Bruijn graph structure is further complicated by palindromic k-mers. In the context of PRICE, de Bruijn graphs were only created in the context of assembly jobs with known relative orientations for all of the input sequences based on paired-end topology.

Their single-stranded nature greatly simplified the construction and evaluation of de Bruijn graphs in PRICE. For each observance of a k-mer in the source data, that k-mer was added to the graph and given/added a score equal to the lowest nucleotide or phosphate score from the observation. Edges between k-mers were given/added the worse of the two phosphate scores linking each k-mer to the additional nucleotide of the adjacent k-mer. K-mers containing ambiguous nucleotides (*i.e.*, N’s) were not included in the graph. Contigs were generated from the resulting graph beginning with the highest scoring node and extending outward bi-directionally, choosing the best linkage to an adjacent k-mer until either a tip was reached or the best-supported edge led to an already-visited node. That process was repeated until no unvisited nodes remained. Thus, the “tip” and “bubble” structures (Zerbino and Birney 2008) that indicate ambiguity in de Bruijn graphs were resolved as short, independent contigs that would either (a) be eliminated due to insufficient coverage scores or insufficient length, (b) be collapsed with the contig generated from the consensus path in a later collapse step, or (c) continue to exist as a distinct sequence in the assembly.

The fourth collapse step combined sequences using ungapped alignments for which one sequence was completely overlapping the other, *i.e.*, was a complete subset. Although not qualitatively different from the fifth collapse step (described in the paragraph to follow), which allowed sequences to overlap only partially, the full-overlap requirement provided numerous algorithmic advantages over the equivalent operation allowing overlaps. These advantages were particularly useful due to the tendency of the de Bruijn graph parsing strategy described previously to generate distinct but nonetheless highly redundant sequences from tips or bubbles.

The fifth collapse step aimed to collapse pairs of sequences sharing high-quality, semiglobal (not penalizing overhangs), ungapped alignments. Potential matches were seeded using perfect matches to sequence substrings. As seeds increase in length and decrease in redundancy, their probability of seeding time-consuming spurious alignments decreases but so does their probability of not seeding what would be a legitimate alignment. Seed sizes and redundancy were selected by PRICE to always maintain a >90% probability of seeding an alignment meeting the input percent-identity requirements. Long subsequences were selected from across each full sequence that could seed alignment to a target sequence with any degree of overlap. Shorter seeds were also selected proximal to the contig termini that were only allowed to seed alignments with shorter overlaps with the target contigs. Perfect matches to all seeds were sought efficiently using a Burrows-Wheeler indexing of the set of all input contigs that was constructed using a linear algorithm (Ferragina and Manzini 2001; Kärkkäinen and Sanders 2003). For each match, the extent of overlap between the two sequences was calculated along with a maximum number of mismatches according to the input percent identity requirement. Seeded alignments were then performed in a fail-fast manner, and all tying highest-score matches were collapsed with the query sequence. To avoid read count amplification in cases where the number of tied high-scoring matches was >1, the nucleotide and phosphate scores from the query contig were appropriately normalized and distributed evenly across the new contigs.

The sixth collapse step repeated the fifth, but now substring matches were used to seed gapped alignments. Gapped alignments were performed using a constrained, fail-fast, semiglobal implementation of the dynamic programming alignment algorithm (the Needleman-Wunsch algorithm (Needleman and Wunsch 1970), modified to not penalize initial gaps and to trace back from the highest-scoring terminal node of either sequence). Seed matches were used to define candidate offsets between sequences, and those offsets were used to define diagonal corridors of permissibility through the dynamic programming alignment grid. The width of each corridor was heuristically set to the square root of the number of gaps that would be allowed given the offset-predicted alignment length and input percent identity requirement for the alignment. Overlapping corridors were merged. Other optimizations were introduced based on the assumption that alignments not meeting the input requirements could be discarded: newly filled squares were evaluated in terms of their accumulated gaps and mismatches to ensure that they could potentially generate a satisfactory alignment. If none of the squares traversed by the optimal path lay along one of the seeded offsets, then the number of gaps required to reach a seeding offset was also considered. Squares incapable of generating a satisfactory alignment were erased from the grid, preventing the spawning of downstream squares. This elimination of unproductive squares reduced the complexity of the dynamic programming task as errors accumulated in the alignment and permitted the alignment to be terminated when a total absence of potentially productive squares in the active portion of the grid revealed that no alignment path could satisfy the input requirements.

### LSV2 assembly

Paired-end sequence data were obtained from Runckel *et al.* (2011) (Dryad repository: doi:10.5061/dryad.9n8rh). Data consisted of 65nt reads. All reads were supplied to PRICE (v0.18; derisilab.ucsf.edu/software/price/); primer random hexamers were not trimmed from the reads. Data were input from paired “_sequence.txt” files (fastq format with Illumina-specific Phred+64 ASCII score encoding) with a maximum amplicon size of 400nt and minimum identity of 90% for mapping to contigs specified with the flag “-fpp [read file 1] [read file 2] 400 90.” Low-quality reads were filtered out by PRICE using the flag “-rqf 90 .95,” specifying that both reads of a pair must have ≥90% of their nucleotides with a ≥0.95 probability of being correct. A single seed sequence deriving from the dataset was provided to PRICE in fasta format: “CACGAGGGCGACAGAATAGAAGACTGCGGCGAGCCTCTGTAACGGGCTGAGTTGGCGGTACTTCA,” using the flag “-icf [filename] 1 1 1.” The assembly was specified to run for 16 cycles using the flag “-nc 16,” although assembly was complete after 12 cycles and the 12th cycle output was used for the analysis presented here. For assembly, minimum sequence overlap of 30nt with a minimum 80% identity were required for collapsing sequences into a contig using the flags “-mol 30” and “-mpi 80,” respectively. Explicit targeting (removal of contigs without apparent similarity to the seed sequence) was invoked with the flag “-target 90 3 1 1,” requiring contigs to share 90% identity with the seed and applying that filter periodically (three cycles off, then every other cycle on). Contigs shorter than the read length were removed after the first cycle with the flag “-lenf 65 1.”

MetaVelvet (Namiki *et al.* 2012) was run on the same input dataset by first using Velvet (Zerbino and Birney 2008) v1.2.08. The velveth command was called specifying a kmer size of 31 and using the –shortPaired and –fastq flags. Then velvetg was called with the following flags: “-exp_cov auto –ins_length 300.” Finally, meta-velvetg was called using the flag “-ins_length 300.”

SOAPdenovo (Li *et al.* 2010b) v1.05 was run on the same input dataset using a config file with the following specifications: max_rd_len = 65, avg_ins = 300, reverse_seq = 0, asm_flags = 3, rd_len_cutoff = 65, rank = 1, pair_num_cutoff = 3, map_len = 32. The variables “q1” and “q2” were then set equal to the two paired-end _sequence.txt files. Assembly was executed with the following command: “./SOAPdenovo-31mer all –K 30 –d 2 –p 80 –R –s [config file] –o [output file prefix].” Demanding an odd Kmer size, SOAPdenovo reported use of –K 31 instead of 30.

The IDBA-UD assembler (Peng *et al.* 2012) was downloaded from i.cs.hku.hk/~alse/hkubrg/projects/metaidba, the website reference in the MetaIDBA publication (Peng *et al.* 2011). The following instruction was provided at the website: “Please note that MetaIDBA is out of maintainance [sic] now, we recommend using IDBA-UD instead which generally performs better.” We followed those instructions, running IDBA-UD 1.1.0 on the same input dataset. First, the data from both paired-end lanes were merged and converted to fasta using the provided “fq2fa” tool. That output file was used as input for the following command: “./idba_ud –r [merged fasta file] –o [output directory].” The “contig.fa” file was used for assembly analysis, although all of the following output files were examined and contained only contigs of equal or lesser quality *vs.* those of the consolidated file: contig-20.fa, contig-40.fa, contig-60.fa, contig-80.fa, contig-100.fa.

Trinity (Grabherr *et al.* 2011) r2012-10-05 was run on the same input dataset using the command “./Trinity.pl–seqType fq –JM 20G–left [first-read _sequence.txt file] –right [second-read _sequence.txt file] –output [output file]–CPU 40.” All assembly commands are provided in File S3.

### Keratin 6A messenger (m)RNA assembly

Paired-end transcriptome data were obtained from accession SRA029929, dataset B, barcode CAC, from Arron *et al.* (2011), corresponding to clinical sample ID STA01-040 (Arron *et al.* 2011). Data were preprocessed as described there to remove barcode, random-primer sequence, and last nucleotide, generating 4,427,628 reads (2,213,814 amplicon pairs) of 54nt each.

Assembly of keratin 6A by PRICE (v0.18; derisilab.ucsf.edu/software/price/) was initiated with a single 54nt read from the aformentioned dataset, found by BLASTn (Altschul *et al.* 1990) to match the reference keratin 6A mRNA (NM_005554.3) with 100% identity, using the flag “-icf [filename] 1 1 0.” That sequence was “TGGCCTCAGCTCTGTTGGAGGCGGCAGTTCCACCATCAAGTACACCACCACCTC.” Paired-end data were included from paired “_sequence.txt” files (fastq format with Illumina-specific Phred+64 ASCII score encoding) with a maximum amplicon size of 400nt and minimum identity of 97% for mapping to contigs specified with the flag “-fpp [read file 1] [read file 2] 400 97.” The assembly was specified to run for 10 cycles using the flag “-nc 10.” Reads pairs with homopolymer tracks (such as poly-A tails) were filtered out using the flag “-maxHp 20.” The minimum overlap and percent identity for collapsing two aligned sequences into a contig were set to 25nt and 90% using the flags “-mol 25” and “-mpi 90,” respectively. Explicit targeting (removal of contigs without apparent similarity to the seed sequence) was invoked with the flag “-target 95 1 4 1.” requiring contigs to share 95% identity with the seed and applying that filter periodically (1 cycle off, 4 cycles on, repeat). The maximum length of sequence to which the de Bruijn method of assembly would be applied was set to 55nt with the flag “-dbmax 55.” Output contigs shorter than 54nt were removed throughout the assembly with the flag “-lenf 54 0.” Assembly was performed on a desktop Macintosh Pro (OSX v10.6.2, 16Gb RAM, 2.26GHz Intel Xeon) using a single CPU core. Command provided in File S3.

### Kaposi’s sarcoma-associated herpesvirus (KSHV) library construction and assembly

KSHV virus was obtained from the primary effusion lymphoma cells BCBL-1 that were grown in RPMI 1640 supplemented with 10% fetal bovine serum, penicillin, streptomycin, glutamine, and β-mercaptoethanol. Virus from BCBL-1 cells was enriched from induced cells after 5 d as previously described (Lagunoff *et al.* 2002; Bechtel *et al.* 2003). Pelleted virus was resuspended in endothelial medium containing 8 μg/mL polybrene, and viral DNA from this medium was obtained as described (Grossmann and Ganem 2008). KSHV DNA was fragmented using a nebulizer and ligated to sequencing/polymerase chain reaction (PCR) adapters according to the instructions for the Genomic DNA Sample Preparation kit (Illumina). The resulting amplicon library was run on a 2% agarose gel, DNA fragments of 250-350 base pairs excised and purified, and size-selected amplicons PCR-amplified for 18 cycles as per the Illumina kit instructions. In preparation for flowcell loading, PCR products were diluted to 100 nM using elution buffer (QIAGEN) and 0.1% Tween 20. Paired-end sequencing was performed on an Illumina HiSequation 2000 instrument for 65nt in each direction (accession SRS367470). The mean length of amplicon inserts (excluding PCR/sequencing adapters) was 207nt (43nt standard deviation), calculated using mapped reads to the reference genomes and excluding 11 outliers >1 kb.

Assembly of KSHV by PRICE (v0.18; derisilab.ucsf.edu/software/price/) was initiated with a fasta file of 65nt fragments taken from even 5-kb intervals across the reference KSHV genome (NC_009333.1) using the flag “-icf [filename] 1 1 2” (seeds provided in Supporting Information, File S1). Paired-end data were included from paired “_sequence.txt” files (fastq format with Illumina-specific Phred+64 ASCII score encoding) with a maximum amplicon size of 500nt and minimum identity of 90% for mapping to contigs specified with the flag “-fpp [read file 1] [read file 2] 500 90.” The assembly was specified to run for 81 cycles using the flag “-nc 81,” although it reached completion by the 65th cycle (Figure 4); output contigs were collected every five cycles using the flag “-nco 5.” Reads were quality-filtered to lessening degrees through the assembly using the flags “-rqf 95 0.998 0 14 -rqf 95 0.99 14 6 -rqf 95 0.9 20 10 -rqf 90 0.9 30 10 -rqf 80 0.6 40 20.” Low-coverage sequence was periodically trimmed from contig termini using the flags “-trim 25 2 -trim 35 2 -trim 45 2 -trim 55 2 -trim 65 3 -trim 70 2.” Minimum output/retained contig sizes were increased through the assembly using the flags “-lenf 60 1 -lenf 70 5 -lenf 80 20.” Contigs that had previously stopped expanding were periodically reset, allowing advantage to be taken of updated cycle-specific specifications, using the flag “-reset 5 10 14 18 20 25 30 35 40 45 50 55 59 60 63 65 70 75.” The explicit targeting option was not used for this assembly. Assembly was threaded to 16 cores using the flag “-a 16.” Assembly was performed on an Ubuntu Linux 12.04 machine (Kernel 3.2.0-39, 64 bit) with four AMD Opteron 6274 CPUs (1.4 Ghz, 16 cores each) and 256 Gb RAM. Sequence complexities for nonoverlapping 250nt genomic windows were determined as the number of additions to the string table during an LZW compression of the DNA sequence (Welch 1984). Command provided in File S3.

## Results

### Implementation of a targeted *de novo* assembly strategy

**Figure 1:**
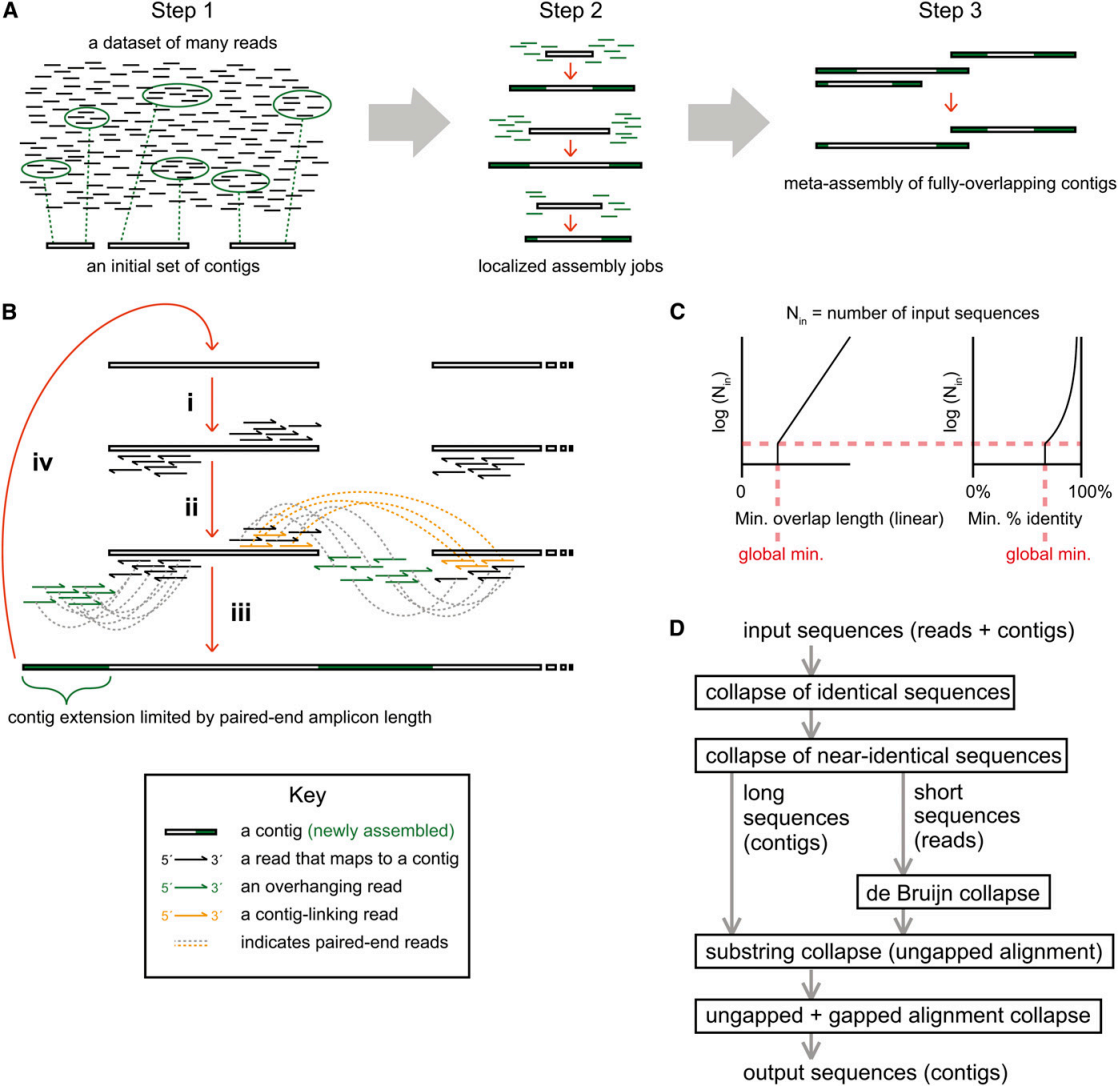
Schematic views of the PRICE assembly strategy. (A) The three general steps of a PRICE assembly cycle: (1) retrieval of reads that are likely to derive from genomic regions proximal to the edges of initial input contigs; (2) localized assembly of each contig with its gathered proximal reads to yield larger, extended contigs; and (3) collapse of highly redundant contigs that were generated in the prior assembly step (meta-assembly). (B) A more detailed description of steps 1 and 2 from (A): (i) read mapping to the outward-facing edges of the input contigs; (ii) gathering of the paired-ends (green) of the mapped reads, along with other input contigs linked by mapped read pairs (orange); (iii) strand-specific assembly of the gathered reads and linked contigs; and (iv) repetition using the output contigs as input for a new assembly cycle. (C) Scaling strategy for assembly requirements (minimum overlap and minimum percent identity between aligned sequences). Both requirements were scaled in proportion to the log of the number of input sequences (y-axis), with the minimum overlap increasing linearly (left x-axis) and the minimum percent identity approaching 100% asymptotically (right x-axis). Global minimum values for both factors were defined and applied at and below a baseline number of input sequences (red dashed lines). (D) Hierarchical workflow for local assemblies using a series of different strategies, with subsequent steps increasing both sensitivity and computational demand. The same steps apply to meta-assembly, but the de Bruijn graph method is not applied in that case and the final gapped and ungapped alignments are limited to cases of extensively overlapping sequences.

Our concept of a targeted assembly strategy involved iteratively repeated execution of three steps: (1) for each seed contig, identification of a subset of data that could be assembled to expand the seed contig; (2) performance of an assembly on each seeded dataset; and (3) elimination of redundancy from the products of individual assembly jobs through performance of a meta-assembly on those products (Figure 1A). Sequences of relevance to the extension of a contig were selected using paired-end read information. Therefore, extension of a contig through a single iteration of this three-step process would be limited to the spacing of the paired-end reads in the source genome (Figure 1B). Arbitrarily long extension of a contig could therefore only be achieved through repetition of the process.

For the clarity of the discussion to follow, paired-end reads will be assumed to derive from opposing genomic strands and to lie 3′ of one another (inward-facing), although PRICE was implemented to also support the outward-facing paired-read topology typical of long-distance mate-pair libraries as well as nonpaired reads that are typically the produced by long-read high-throughput sequencing technologies. In the former case, so-called mate-pair reads do not differ qualitatively from paired-end reads, but that term is typically applied to libraries where the two reads derive from relatively distant loci in the reference genome (multiple kilobases). The construction techniques for such libraries often result in read orientations different from those of typical paired-end libraries. In the latter case, PRICE can use single sequences either as seed contigs or can split them into variably overlapping, artificial paired-ends.

For the first step of the PRICE assembly strategy, the identification of a subset of read data that could contribute to the extension of each contig, the entire paired-end dataset was mapped to the seed contigs (Figure 1Bi), the goal being identification of the paired-end reads deriving from the same parts of the source genome as the termini of the seed contigs. From that perspective, reads whose map positions were distant from the contig 3′ termini (the direction of the paired-ends) were irrelevant to extension of the contig. Initial read mapping was therefore limited to being within a user-defined distance of the 3′ terminus of each strand of the seed contig. This limitation greatly reduced the complexity of the mapping task, making it scale approximately with the number of seed contigs rather than the total size of the assembly.

In some cases, both reads of a pair would map, each to the terminus of a distinct contig (Figure 1Bii, orange). This suggested the adjacency of those two contigs in the source genome, with the amplicon sizes of the library further suggesting an approximate intervening distance. In that case, each read would be replaced with the contig to which it mapped in the assembly job for each of the seed contigs. If the majority of contig-linking reads from each of those two contigs connected them (>50%, with all read counts normalized to the number of contigs to which they were mapped), then their assembly jobs would be fused into a single nonredundant job. For extensively overlapping contigs, the possibility existed that only one read of a pair would map to a contig terminus. All terminus-mapped reads and their paired ends were therefore re-mapped to the entire assembly. The reduction in complexity of the mapping job due to the small fraction of reads that were initially mapped to contig termini would generally compensate for the increase in complexity due to the inclusion of the entire current assembly as a “mappable” target (a target/region to which reads can be accurately mapped with a given informatic tool).

Remapping of reads also provided an opportunity for critical reassessment of the original mappings to 3′ contig termini. If reads could be aligned to an interior position, or one proximal to a 5′ contig terminus, with a superior score *vs.* that of the original alignment, then the new mapping position replaced the old (for a description of the scoring system based on the ratio of supporting *vs.* conflicting input data, see *Materials and Methods*). If both reads of a pair were displaced by new mappings, then that read pair was removed from the extension/linkage dataset. At this stage, the alignments of reads deriving from repetitive genomic elements were also invalidated. PRICE implemented three techniques for detecting such sequences: (1) alignment to a user-provided database of repetitive element sequences; (2) alignment to a similar database generated by PRICE during assembly based on regions of anomalously high read coverage; and (3) equally scoring alignments to multiple distinct initial contigs.

The second step of the PRICE assembly strategy, performance of an assembly job for the collection of sequences associated with each initial contig (Figure 1Biii), was designed to dynamically balance efficiency and sensitivity. Efficiency was favored for jobs with many input sequences, suggesting high coverage that will be sufficient to create an accurate assembly even with high stringencies for alignment. Sensitivity was favored for jobs with few input sequences, suggesting low coverage that would necessitate low alignment stringencies in order to produce a successful assembly but would also imply a low probability of spurious misassembly. Assembly requirements, in terms of minimum alignment/overlap length and minimum percent identity match for the alignment, were scaled covariantly with the number of sequences in the assembly job, *i.e.*, inversely with the naïve probability of there being an alignment between two sequences in the assembly job that was generated by chance (Figure 1C).

A variety of strategies can be used to collapse redundant input sequences into contiguous consensus sequences, including the simple collapse of identical sequences, the use of de Bruijn graph representation (Zerbino and Birney 2008) to generate a consensus view of overlapping sequences, and more time-consuming binary alignment-then-collapse methods. For PRICE, the following strategies were implemented and executed in series, with subsequent collapse steps executing assembly strategies of increasing computational complexity and sensitivity (Figure 1D; see *Materials and Methods* for details): (1) the collapse of identical sequences, (2) the collapse of near-identical sequences (equal in length and aligned without gaps or offset), (3) de Bruijn graph-based assembly (limited to the short sequences), (4) pairwise alignment and collapse using ungapped alignments for fully-redundant sequences, and finally (5/6) pairwise alignment and collapse for overlapping sequences, first using ungapped, then gapped, alignments.

Although PRICE was not built to output scaffold structures, such structures were built internally during the assembly process. The scaffolding performed by PRICE consolidated series of paired-end-linked contigs into single assembly jobs. The scaffolding requirements set by PRICE were generally much less stringent than those imposed by dedicated scaffolding software or the scaffolding components of other genome assemblers (Pop *et al.* 2004; Butler *et al.* 2008; Li *et al.* 2010b), but scaffolding only contributed to PRICE output if validated by common, overlapping sequences at the contig termini that allowed the joining of the linked contigs into a single sequence.

The third step of our assembly strategy, the elimination of redundancy in the final contig set through performance of a meta-assembly (Figure 1A), had an implicit efficiency advantage over the seeded assembly jobs deriving from its definition. In order for two sequences to be truly redundant, the area of the source genome covered by one sequence must be a subset of that covered by the other. Therefore, a redundancy collapse required all alignments to be global with respect to one of the two sequences and semiglobal with respect to the other. Although the complexity of the meta-assembly would still scale with the number of contigs in the full assembly, the complexity of comparison for any two sequences was reduced to the difference between their lengths rather than the sum of their lengths. In practice, this kept meta-assembly jobs manageable, even when large (multi-kilobase) sequences were being compared using gapped alignments and even without the application of the de Bruijn graph strategy that PRICE reserved for the short sequence components of the small, strand-specific, contig-centered assembly jobs.

### Applications of PRICE to diverse biological problems and datasets

PRICE is free and open-source software, and early alpha-stage implementations were made available to the broader research community through our website (derisilab.ucsf.edu/software/price/ or sourceforge.net/projects/pricedenovo/). We were able to evaluate the utility of PRICE during development in terms of its ability to contribute to real-world projects involving targeted analysis of genomic or metagenomic sequence datasets. The full assembly protocol described previously was the product of iterative redesign to address challenges encountered during these applications. Below, the contributions made by PRICE to published discoveries are reviewed. These include the sequencing of novel viral genomes from metagenomic datasets and the targeted assembly of genes-of-interest from whole-genome datasets.

**Figure 2:**
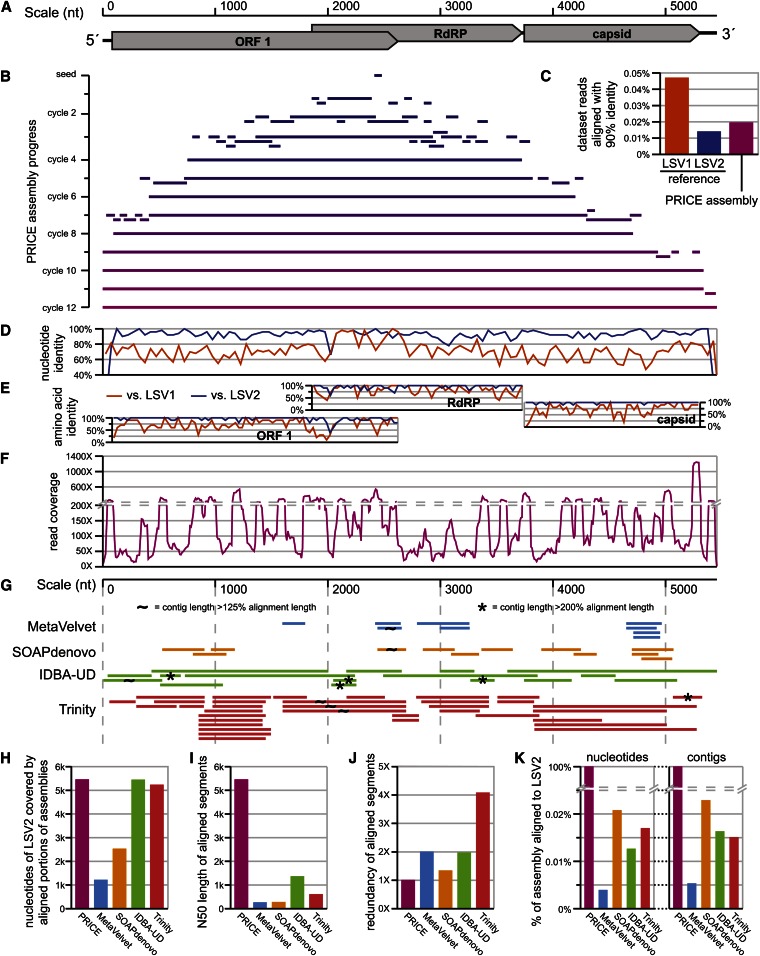
*De novo* assembly of an RNA virus genome from a metagenomic dataset. (A) Scale and genic structure of LSV2, a positive-strand RNA genome encoding three ORFs: ORF1 (function unknown), RdRP, and capsid (Runckel *et al.* 2011). (B) Assembly of LSV2 by PRICE seeded with a single 65nt read. Contigs from each step of a 12-cycle PRICE assembly aligned to the single 12th-cycle output contig. (C) Percentage of reads from the full input dataset that could be aligned to the GenBank reference LSV1 (HQ871931) or LSV2 (HQ888865) genomes, requiring ≥90% identity across the entire read length. (D) Nucleotide % identity of nonoverlapping 50nt windows of the PRICE-assembled LSV2 *vs.* the reference LSV1 (orange) and LSV2 (blue) genomes. (E) Amino acid % identity of nonoverlapping 10aa windows across each of the three LSV ORFs (starts and ends defined by the reference LSV2 annotations) *vs.* the protein sequences for ORF1/RdRP/capsid from LSV1 (orange; AEH26192/AEH26193/AEH26194) and LSV2 (blue; AEH26187/AEH26189/AEH26188). (F) Read coverage across the PRICE-assembled LSV2 genome. Coverage values are averaged across nonoverlapping 10nt windows. (G) Contigs from assemblies performed on the same paired-read dataset as above (Dryad repository: doi:10.5061/dryad.9n8rh) using MetaVelvet (Zerbino and Birney 2008; Namiki *et al.* 2012) (blue), SOAPdenovo (Li *et al.* 2010b) (orange), IDBA-UD (Peng *et al.* 2012) (green), and Trinity (Grabherr *et al.* 2011) (red). Bars indicate alignments between contigs output by those assemblers and the PRICE-assembled LSV2 generated by BLASTn (Altschul *et al.* 1990) and covering ≥150nt on the PRICE LSV2. Analysis was limited to contigs ≥200nt. Contigs are marked whose lengths are >125% (~) or >200% (*) that of their aligned segments. (H) PRICE sensitivity: the number of nucleotides from LSV2 encompassed by the alignments shown in (G) for each assembly. I) The N50 length for alignments shown in (G). (J) The redundancy of the aligned portions of each assembly shown in (G). Calculated as the summed lengths of the aligned segments divided by the length of their total footprint on the LSV2 assembly from (H). (K) PRICE specificity: the % of nucleotides or contigs from each assembly that were aligned to LSV2 in (G). Chimeric contigs that only partially aligned to LSV2 were fully counted. Only contigs ≥200nt were considered for both the alignments and the total assembly size.

The motivation for PRICE was to facilitate the assembly of individual genomes of interest using complex, shotgun, metagenomic datasets, derived from virtually any origin, be it clinical or environmental. As source code was made publicly available before the writing of this manuscript, several examples of genome assembly using PRICE have been published (Runckel *et al.* 2011; Grard *et al.* 2012; Holmes *et al.* 2012; Kaysser *et al.* 2012; Stenglein *et al.* 2012a,b; Melters *et al.* 2013). The first demonstration of PRICE’s utility for assembly of novel viral genomes was in the context of a survey of the pathogens that infect honey bees in the United States (Runckel *et al.* 2011). For that study, sequence data were collected from nucleic acid isolated from multiple whole bees, collected from different hives and carrying different pathogens. The source data set was exceptionally complex, containing data derived from the host genome (*Apis melifera*), the genomes of the bees’ symbiotic microflora, and the genomes of a variety of cellular and viral pathogens, including *Spiroplasma sp*., *Crithidia sp*., *Nosema sp*., and a multitude of RNA viruses (Runckel *et al.* 2011). Alignments of the short reads to known viral genomes, followed by extension of those sequences into larger contigs using PRICE, revealed the genome sequences of four novel viruses: two *Dicistroviridae* (Aphid Lethal Paralysis virus strain Brookings and Big Sioux River virus) and two *Nodaviridae* (Lake Sinai Virus strain 1 and 2; LSV1/2). The assembly of LSV1/2 made for an informative comparison relative to other assembly algorithms, the details of which are presented in the following section and in Figure 2.

Similar targeted assembly approaches have been used to obtain full genome sequences for other animal viruses, namely a rabbit *Astroviridae* (Stenglein *et al.* 2012b), as well as two novel *Arenaviridae* that infect snakes (Stenglein *et al.* 2012a). In the latter case, the identified viruses are highly divergent members of a viral family otherwise only known to infect mammals. Their discovery was aided by the filtering away of host sequence using *Boa constrictor* genomes generated for the second iteration of the Assemblathon genome assembly competition (assemblathon.org) (Earl *et al.* 2011). Most recently, PRICE has been used to recover the genome of a *Flaviviridae* associated with Theiler’s disease (hepatitis) in horses (Chandriani *et al.* 2013) . In addition to these applications to veterinary medicine, PRICE has also been used to address human infectious diseases, namely through the assembly of a novel and divergent rhabdovirus isolated from a human patient with hemorrhagic fever (Grard *et al.* 2012).

An alternative application for PRICE was the targeted assembly of genomic regions of interest from single-species datasets. This single-genome application of PRICE has been applied in two contexts of note. In the first context—the discovery of centromeric tandem repeat elements from diverse plant and animal species—the high apparent coverage and polymorphism of these elements due to their high copy numbers in the genome made them recalcitrant to assembly using typical whole-genome assemblers (Melters *et al.* 2013). When independently seeded by randomly selected reads, PRICE could treat these repeats as if they were independent, high-copy-number components of a metagenomic mixture and assemble a consensus element, thereby revealing their structures and evolution across diverse taxa (Melters *et al.* 2013).

Targeted assembly from single genomes, limited to regions of interest, has also been applied to the discovery of natural product biosynthesis pathways, such as those that generate antibiotics in diverse bacteria. The genes needed to synthesize antibiotics from common biological metabolic precursors are often clustered in bacterial genomes, accompanied by genes that confer antibiotic resistance to the host cell; such an arrangement increases the probability of the entire synthesis machinery being retained on a single fragment of sheared DNA and thereby increases the fitness of that DNA in the context of all of the major horizontal gene transfer mechanisms (transformation, bacteriophage transmission, and conjugation) (Lawrence and Roth 1996; Dobrindt *et al.* 2004). PRICE was therefore applied to the initial assembly of the biosynthesis gene cluster for the merochlorin A-D meroterpenoid antibiotics of *Streptomyces* sp. CNH-189 (Kaysser *et al.* 2012). In that case, structural knowledge of the antibiotics was used to generate hypotheses of enzyme-catalyzed reactions that might be involved in the synthesis of the CNH-189 merochlorins, and seed sequences from CNH-189 were sought by aligning translated reads to known proteins that catalyze similar reactions from other bacteria. Extension by PRICE of the few reads with high-confidence alignments into larger contigs revealed the full genes from which those reads derived, alongside surrounding genes that provided additional information about the gene content of the cluster and allowed contigs built from spurious matches to the hypothetically homologous enzymes to be confidently dismissed. Targeted assembly by PRICE was used in a similar manner to uncover the biosynthetic pathway for the guadinomine family of antibiotics produced by *Streptomyces* sp. K01-0509 (Holmes *et al.* 2012).

The aforementioned examples encompass a variety of dataset types, assembly challenges, and research goals. In the sections to follow, we present three additional sample assemblies to highlight the ability of PRICE to address particular assembly challenges commonly encountered in real-world datasets. The first was a repetition of the LSV2 assembly described previously, presented in tandem with the results obtained using other assembly software applied to the same dataset. The second, assembly of a keratin mRNA from a transcriptome dataset deriving from a human cancer sample, illustrated the ability of PRICE to both accommodate highly uneven coverage that is typical of sequenced RNA (RNAseq) libraries, especially those that are constructed using mRNA-enrichment techniques, and to remain focused on a sequence of interest even when erroneous data suggests that off-target assemblies should be performed. And the third, assembly of a previously unsequenced strain of the KSHV, illustrated the utility of PRICE for *de novo* assembly of nucleic acid sequences significantly longer than mRNAs, as well as its ability to remain focused on target sequences even through the many cycles of contig extension (and many opportunities for off-target assembly) required by such an assembly.

### Specific assembly of an RNA virus genome from a complex metagenomic dataset

As described previously, PRICE was used to obtain complete genome sequences from a complex metagenomic mixture of nucleic acid associated with honeybees (Runckel *et al.* 2011). Here, we provide details of a reassembly of one of those viral genomes, that of LSV2, from that metagenomic dataset (Dryad repository: doi:10.5061/dryad.9n8rh). This dataset consists of 65.1M 65nt paired-end reads (32.5M pairs). The source nucleic acid mixture derives from all 20 of the hives being monitored for the study, with 50-100 individual bees per hive, with libraries prepared following four separate protocols, three of which could have captured purely RNA species such as the LSV2 virus. The resulting dataset includes a diverse mixture of sequences from not only the bees themselves, but also from the full diversity of their natural microbiomes (gut and surface) and a panoply of viral and nonviral pathogens (Runckel *et al.* 2011).

The ~5.5-kb RNA genomes of the Lake Sinai viruses contain three open reading frames (ORFs), encoding a protein of unknown function (ORF1), an RNA-dependent RNA polymerase (RdRP), and a capsid protein (Figure 2A), with the ORF1 and RdRP encoded by overlapping, offset frames. The genome and proteome sequences are particularly distant from those of previously known viruses, sharing only 25% amino acid identity with their closest known relative in the most highly conserved ORF (the RdRP) (Runckel *et al.* 2011). Such sequence divergence obfuscates the retrieval of reads based solely on homology, shifting the burden for whole genome sequence recovery onto the shoulders of the assembler. Matters were further complicated by the presence of two distinct but similar strains of Lake Sinai virus in this dataset (LSV1).

For the sample assembly shown in Figure 2B, we selected a single 65nt read from the RdRP gene of LSV2 as a seed. The complexity of the dataset was highlighted by the tiny fraction of reads that could be aligned to the reference LSV2 genome (Figure 2C). Inclusion of the random-hexamer-derived nucleotides from the library primers further obfuscated the correct nucleotide identities of the genome: unpaired nucleotides from the original priming event generate 5′ sequences with accurately called nucleotides that nonetheless do not match the reference genome. Assembly was performed for 12 cycles, producing a single contig of 5456 nt. Though knowledge of LSV derives from PRICE assemblies of this dataset, the reference LSV1 and LSV2 genome sequences (HQ871931 and HQ888865, respectively) derive from Sanger sequencing of reverse transcription PCR products amplified from a separate sample (Runckel *et al.* 2011). This assembly and the reference sequence were therefore likely to derive from similar yet distinct viral strains. Overall, the nucleotide percent identity of the new assembly *vs.* the reference genome was high (Figure 2D), and the amino acid percent identity for the LSV2-encoded proteins was higher (Figure 2E), suggesting that the assembly was largely correct. The new assembly was also consistently closer to the reference LSV2 sequence than the reference LSV1 sequence (Figure 2D-E) despite the presence of far more LSV1-matching reads in the dataset (Figure 2C), further illustrating the ability of PRICE to remain focused on a sequence of interest, avoiding chimeric aberrations, even in the presence of related sequences.

Read coverage of LSV2 was dramatically variable across the length of the genome (Figure 2F). This consequence of biases in the library preparation methods can pose a challenge to genome assembly. The built-in scaling of assembly overlap requirements by PRICE to reflect local coverage was intended to address these biases. Further, the local nature of PRICE assembly jobs (limitation of input sequences to those inferred to derive from local genomic regions based on paired-end information) was intended to avoid the chimeric misassemblies that would be expected to arise when applying reduced alignment stringency to a full dataset. We assessed the potential advantage provided by PRICE over competing assembly strategies in performing assemblies of metagenomic components from large datasets with the confounding factors observed here: the scarcity and unevenness of relevant data. Assemblies were performed using four other software packages: MetaVelvet (Namiki *et al.* 2012), a metagenome-optimized variant of the de Bruijn graph assembler Velvet (Zerbino and Birney 2008); the de Bruijn graph assembler SOAPdenovo (Li *et al.* 2010b); the metagenome-optimized assembler IDBA-UD (Peng *et al.* 2012); and the transcriptome assembler Trinity (Grabherr *et al.* 2011) (see *Materials and Methods* for details). Alignments of output contigs from those assemblies to the LSV2 genome assembled by PRICE are shown in Figure 2G.

Two distinct failure modes were apparent from the performance of the four other assemblers. The first applied to MetaVelvet and SOAPdenovo. These assemblies were characterized by a small number of short contigs that appeared to derive from LSV2 (Figure 2G). The LSV2 contigs covered small portions of the LSV2 genome, *i.e.*, exhibited low sensitivity (Figure 2H), and individually covered only short fragments (Figure 2I). The second failure mode applied to IDBA-UD and Trinity and was characterized by extensive coverage of the LSV2 genome (Figure 2H) and longer assembled fragments (Figure 2I), but also by high redundancy, with many contigs containing slight sequence variations repeatedly covering the same parts of the genome (Figure 2J). Both failure modes included chimeric contigs with a significant portion of their sequences deriving from a non-LSV2 source, but these mis-assmblies were more commonly observed in the second failure mode, in which the LSV2 contigs were more numerous (Figure 2G).

The targeted assembly strategy implemented by PRICE was not only intended to improve assembly of the target sequence, but was also intended to focus post-assembly analysis on a limited number of sequences that were selected by the investigator to be “of interest” before assembly and provided as seeds, *i.e.*, provide specificity of assembly. The LSV2 dataset used here included scarce quantities of LSV2-relevant data (Figure 2C). The output of the non-PRICE assemblers reflected this paucity of LSV2 data, with only a small portion of the assembly output deriving from LSV2 in each case (Figure 2K).

### Transcript assembly in the context of highly uneven coverage

Transcriptomes are analogs of metagenomes, with the mRNA product of each gene being analogous to an organismal species within an ecological community and with each mRNA molecule analogous to an individual of that species in the ecosystem. Like species in metagenomes, some transcripts are much more abundant than others, with distinct genes sharing varying degrees of interrelatedness and possibly differing in the magnitudes of their own intrinsic sequence polymorphism.

**Figure 3:**
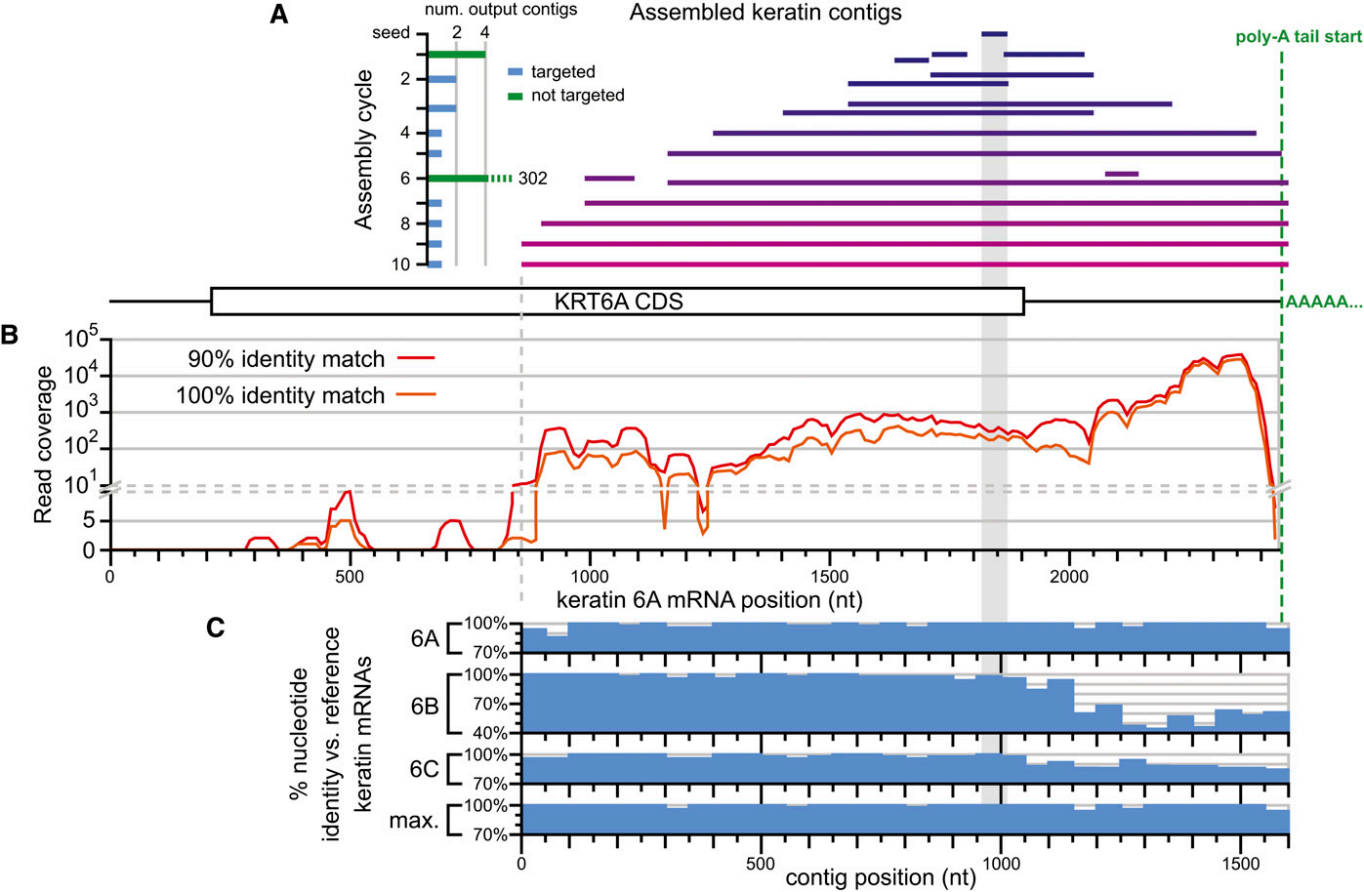
*De novo* assembly of the keratin 6A mRNA from a transcriptome dataset. (A) Contigs from each step of a 10-cycle PRICE assembly aligned to the keratin 6A reference sequence (NM_005554.3) by BLASTn (Altschul *et al.* 1990). The seed sequence is a single 54nt read from the paired-end transcriptome dataset (Arron *et al.* 2011) (dark blue; see *Materials and Methods*); the later contigs (purple) include poly-A tail sequence not included in the reference sequence. Left: the total number of output contigs generated in each cycle, shown as a histogram of blue or green bars for cycles that were or were not explicitly targeted (using the –target flag; see *Materials and Methods*) to the seed sequence, respectively. (B) Read coverage from the 54nt paired-end read dataset determined by mapping to the keratin 6A reference. Units are the number of reads overlapping each nucleotide, averaged across nonoverlapping 10nt windows. Coverage is shown requiring 90% (red) or 100% (orange) nucleotide identity between the read and the reference. (C) Identity of the PRICE contig *vs.* the reference keratin 6A sequence, as well as the human keratin 6B and 6C isoforms (NM_005555.3 and NM_173086.4, respectively) for nonoverlapping 50nt windows and including the poly-A tail sequence. Bottom, the maximum % identity for each 50nt contig window to the three keratin 6 isoforms.

To illustrate *de novo* transcript assembly, PRICE was used to assembly the mRNA sequence of human keratin 6A using a published paired-end transcriptome dataset from a cutaneous squamous cell carcinomatumor of the keratoacanthoma subtype (NCBI Sequence Read Archive accession SRA029929; Figure 3) (Arron *et al.* 2011). The dataset included 2.2M amplicons, each sequenced from either end to generate 4.4M reads of only 54nt (after the removal of barcodes and random-primer nucleotides; see Arron *et al.* 2011 for details). This assembly highlighted several features of PRICE, primarily its ability to accommodate hugely inconsistent read coverage levels, even across a single transcript. The library in question derives from random-hexamer priming of RNA templates, but those RNA’s were generated from poly-A−enriched cellular RNA that was further amplified using *in vitro* transcription driven from the 3′ end of the original mRNA (Arron *et al.* 2011). This approach both enriches for the 3′ portions of any fragmented RNA’s from the original samples and generates abortive transcripts whose sequence is biased toward the 3′ end of the original mRNA. Combined, those techniques substantially biased the coverage toward the 3′ end of transcripts.

For keratin 6A, although the 3′ end of the transcript had >10,000-fold coverage, most of the transcript was covered at ~100-fold, and a substantial 5′ portion, including much of the CDS and all of the 5′ UTR, had zero coverage (Figure 3B). The PRICE-generated contig of keratin 6A extended to within a read’s length of transcript’s fold-coverage reaching zero (Figure 3A-B, dashed gray line). At the 3′ end, the contig extended all the way to and included the poly-A tail (Figure 3A, dashed green line).

Assembly of keratin 6A was further hampered by the presence, in the same dataset, of two closely related paralogous transcripts: keratin 6B and keratin 6C. For much of these transcripts, their percent nucleotide identity with one another exceeded the thresholds used by PRICE for redundant contig collapse, resulting in the construction of a consensus sequence that generally favored the 6A isoform of keratin (Figure 3C). However, the single output contig was composed entirely of keratin mRNA sequence, with no additional contigs deriving from other transcripts. This focus was provided by an explicit targeting feature built into PRICE that eliminates contigs with no discernible similarity to the initial seed sequences.

In paired-end RNAseq datasets, template switching by reverse-transcriptase can generate chimeric read pairs that combine sequences from distinct transcripts (Roy and Irimia 2008). Such chimeric read pairs provide data that spuriously support off-target assembly, as was observed in the form of explosive expansion of the number of contigs generated during those few assembly cycles that were not explicitly targeted (cycles 1 and 6, Figure 3A). This transcript assembly thereby illustrated the capacity of PRICE to remain focused on the pre-determined goal of the assembly, even in the presence of data erroneously supporting off-target assembly, using the explicit targeting feature.

### Assembly of a large DNA virus (KSHV) from a multigenome dataset

**Figure 4:**
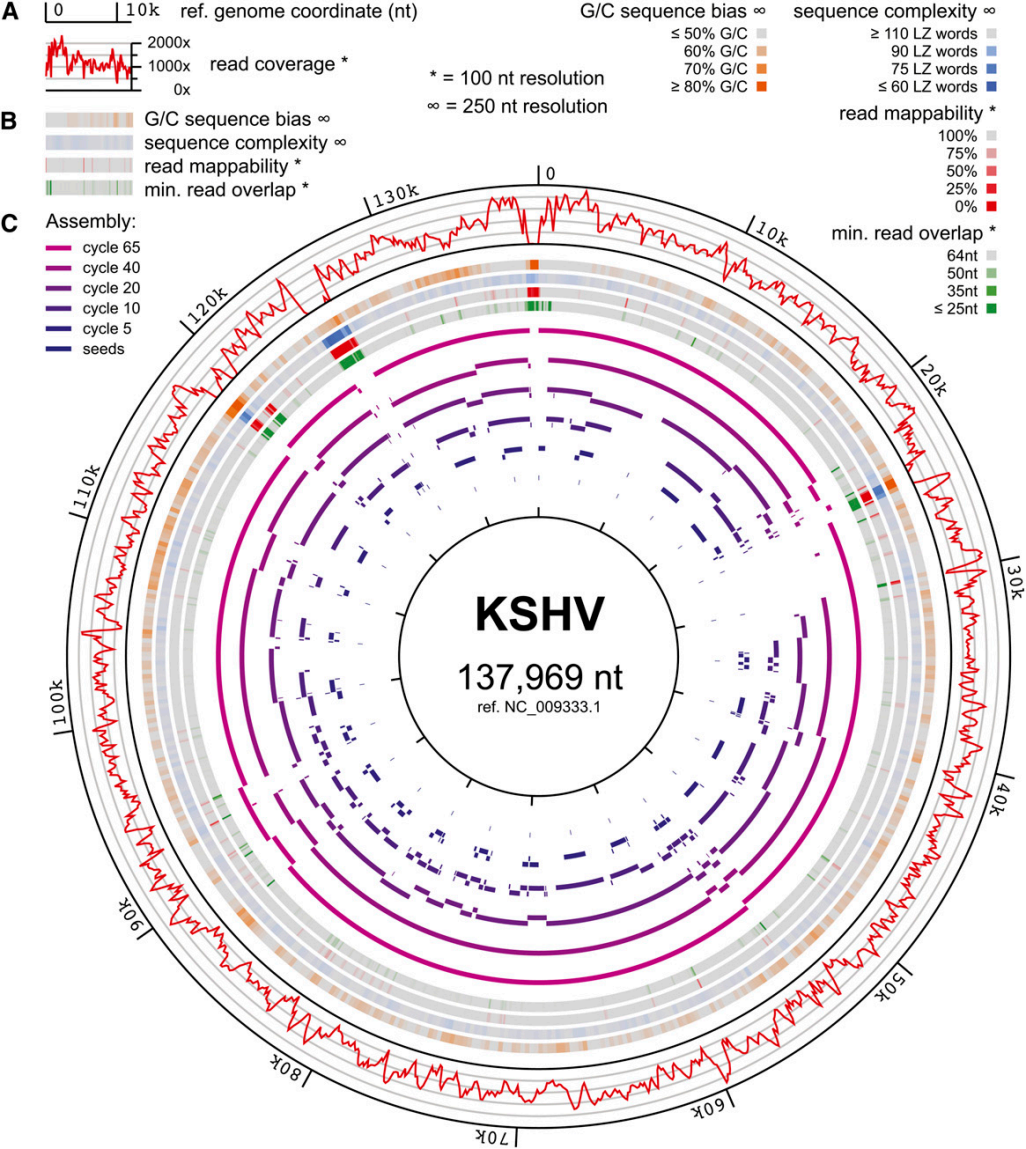
*De novo* assembly of the BCBL-1 strain of the KSHV. (A) Read coverage from the 65nt paired-end read dataset determined by mapping to the KSHV reference genome (Rezaee *et al.* 2006) (NC_009333.1) with BLASTn (Altschul *et al.* 1990), requiring 90% identity. Units are the number of reads overlapping each nucleotide, averaged across nonoverlapping 100nt windows. (B) Heat maps indicating the percent of nucleotides that are G/C in nonoverlapping 250nt windows (orange), LZW sequence complexity (Welch 1984) of nonoverlapping 250nt sequences (blue; see *Materials and Methods*), read mappability as determined by mapping every overlapping 65mer from the genome using the same method as in (A) and averaging the coverage over a 100nt window (red), and the minimum overlap between adjacently mapping reads across each 100nt window, measured as the minimum value across all reads with 3′ ends in the window, measuring the maximum overlap with all reads mapped 3′ of the given read (green). (C) Contigs from selected steps of a 65-cycle PRICE assembly aligned to the reference genome. Seed sequences of 65nt are shown as the innermost ring (dark blue), followed by intermediate contigs aligned to the reference genome by BLASTn (Altschul *et al.* 1990), with the final contigs aligned to the reference genome by the Smith-Waterman method (Smith and Waterman 1981) shown on the outer ring (purple).

The examples of PRICE assemblies described and referenced previously focused on fragments of sequence rarely exceeding a few kilobases in length, and for which the primary challenges facing assembly reflected quality of the input dataset, uneven coverage in particular. However, the same localized approach to assembly can be applied to larger genomes, either by seeding with arbitrary portions of the genome (*i.e.*, using randomly-selected reads) or by focusing on portions of the genome of particular interest (similar to the approaches above, but using seeds from many parts of the genome in parallel). To illustrate this approach, we constructed a dataset from and performed an assembly of the genome of the KSHV (Figure 4). This paired-end dataset (NCBI Sequence Read Archive accession SRS367470), with 65nt reads from each end of each amplicon, derived from the BCBL-1 KSHV-infected cell line (Renne *et al.* 1996), and although it contained very high coverage of the KSHV genome (~1100-fold; see *Materials and Methods*), only a small minority of the reads derived from KSHV (4.4%). The remainder primarily derived from the human host cells (92.3% of all read pairs had at least one read that could be aligned to the reference human genome with at least 90% identity, combining alignments found by BLAT (Kent 2002) and BLASTn (Altschul *et al.* 1990)). This assembly therefore also tested the ability of PRICE to ignore a large amount of irrelevant genomic data when performing a targeted assembly of a large viral genome.

The DNA genome of KSHV enabled construction of a library with consistent coverage across the genome (Figure 4A) *vs.* the RNA-derived datasets described previously. Remaining regions of depleted coverage could have been explained by either or both of two models: (1) amplicon depletion due to PCR bias or (2) apparent but untrue lack of coverage due to mappability bias. Regarding the first model: amplicons from regions of high G/C nucleotide bias can become depleted during PCR amplification of libraries such as these (Aird *et al.* 2011). However, regions with high G/C content (Figure 4B) were generally represented well in our dataset (Figure 4B). Regarding the second model: the multiplicity of equally valid mapping positions for sequences repeated in a genome obfuscates their correct assignment to a genomic region, making reads from such regions less “mappable,” both intrinsically and due to the processes by which software searches for significant alignments. In particular, the lowering of sequence complexity increases the probability of multiple, equally good alignments, but low complexity is not the only property that can cause mappability problems. In the case of KSHV, regions of apparent extremely low coverage often coincided with regions of diminished sequence complexity, independent of the G/C complexity of those regions, and always coincided with regions of low mappability (Figure 4B), indicating that data from such regions were not necessarily depleted in the dataset.

Our assembly of KSHV began with 28 seeds selected at even 5-kb intervals across the KSHV reference genome (File S1) (Rezaee *et al.* 2006) and proceeded for 65 cycles of PRICE extension to yield 13 contigs covering 97.3% of the reference genome (File S2; Figure 4C). In support of the hypothesis that unmappable reads were present in the dataset, many of the regions with apparent low coverage due to low mappability were correctly traversed by the assembled contigs (Figure 4C, positions ~30k, ~93k, ~120k), whereas regions whose unmappable regions substantially exceeded the calculated mean amplicon size of 207nt remained largely unaccounted for in the assembly (Figure 4C, positions ~0, ~25k, ~119k, ~126k). In some cases, contigs abutted one another without being joined despite apparently high read coverage. However, all of those cases corresponded to positions where adjacent reads overlapped by only ~25nt, the specified minimum overlap allowed between two sequences for collapse into a contig for our assembly (see *Materials and Methods*; Figure 4B, positions ~23k, ~54k, ~88k, ~91k, ~94k).

This assembly of KSHV illustrated two strengths of PRICE. First, despite the majority of the dataset deriving from the human genome of the host cells in which KSHV was cultured, the assembly remained focused on only the KSHV component of the dataset. In the keratin assembly described previously, the targeting feature of PRICE was used to eliminate contigs with little discernible similarity to the seed sequence. This avoided incorporation of misleading chimeric data that can derive from reverse-transcriptase template-switching (Roy and Irimia 2008). However, the explicit targeting feature was not used for the KSHV assembly (see *Materials and Methods*). KSHV remained the sole subject of successful assembly through a large number of cycles and through iterative, stepwise growth across many kilobases of novel genome sequence. Consideration of this result in conjunction with the use of explicit targeting for the keratin assembly illustrated a low propensity for PRICE to spuriously engage in off-target assembly work not supported by data.

Although the correction of sequence data are generally inappropriate for metagenomic analysis, PRICE did implement a feature that filters input data based on sequence quality scores in a user-controllable and dynamic manner. This feature was used for the assembly of KSHV. In principle, stringent quality filtering should improve both the quality and efficiency of the assembly, but such filtering also has the potential to prevent assembly through low-coverage regions. We therefore took advantage of the dynamic aspect of PRICE quality filtering subjecting early cycles to very stringent quality requirements, followed by less stringent requirements in later cycles to fill in low-coverage regions (see *Materials and Methods*).

## Discussion

### Genome *vs.* metagenome assembly

The drastically lower cost of nucleic acid sequencing provided by modern high-throughput technologies permits a paradigm shift away from *in vitro* and toward *in silico* enrichment techniques. PRICE was designed to facilitate this transition by enabling convenient targeted assembly of metagenomic subcomponents of interest. Previously implemented genome assembly tools have been designed assuming that all or most of the data collected is relevant to the scientific goal, and that has been an appropriate design choice because it has been the case for genome sequencing projects to date. However, especially for metagenomic datasets, an investigator may only be concerned with individual genes or genomes, rendering wasteful any effort spent assembling data from other aspects of the data.

In addition, several of the assumptions that can be made about single-genome datasets that facilitate efficient assembly become irrelevant in the metagenomic context. First, the read coverage across a single genome will be highly consistent, excepting for variation due to statistical sampling error and known biases based on nucleotide content (Lander and Waterman 1988; Aird *et al.* 2011). Repetitive sequence elements, whose multiplicity within a genome obscures the appropriate pairwise matching of flanking sequences, reveal themselves by their apparent anomalously high read coverage when considered as single-copy elements, allowing assemblers to avoid generating erroneous assemblies around them. This assumption of consistent coverage is irrelevant for metagenomic datasets, where the level of coverage for each genome will be different and dictated by the number of cells and genome copies of each organism in the locally sampled ecosystem. Although PRICE did provide an optionally invokable tool for detecting and masking repetitive elements on the basis of anomalously high read coverage, its default behavior was to not apply such a strategy; that would be the appropriate behavior for metagenomic data.

A second assumption made of genomic data that is irrelevant to metagenomic analysis is the rarity and consistent nature of true sequence polymorphism within a sample. The ratios of coverage between genomes from different species or strains/subspecies in an ecosystem sample span a continuum, unlike the quantized levels of coverage determined by ploidy or repeat copy-number differences. The quantized distribution of coverage among single-genome data simplifies the distinction between true polymorphism and sequencing errors: true polymorphisms will occur at frequencies whose denominators match the ploidy of the sequence element (for commonly-sequenced diploid species, that denominator is only two), whereas errors will occur at much lower frequencies *vs.* the consensus. In metagenomic data, the continuous ratios of polymorphism make such distinctions theoretically impossible, blurring the line between a correct sequence for one species and a consensus sequence for several. Such ambiguity between sequencing errors *vs.* true polymorphisms is especially relevant to viruses with RNA genomes, whose genomes rapidly accumulate polymorphisms and exist as a population of diverse quasi-species, even in the context of single infections (Lauring and Andino 2010). Therefore, PRICE was built to seek a consensus sequence, again favoring behavior appropriate for metagenomic data.

In terms of algorithmic efficiency, the strategy for targeted assembly of metagenomic components that was implemented by PRICE scales differently *vs.* whole-genome assembly algorithms. These differences represent trade-offs that could be advantageous or disadvantageous depending on the context. The principle disadvantage of the PRICE strategy was the requirement of read mapping to contig edges in every cycle. The cost of this requirement grew linearly with the size of the paired-read dataset, the number of contigs being extended (but not their length, as the mappable area is defined by paired-read amplicon size), and the number of cycles run (desired size of output contigs). The advantage gained was the limit of individual assembly jobs to locally relevant reads, typically a minor fraction of the total dataset.

Genome assembly by the traditional overlap-layout-consensus method is a polynomial process, requiring each sequence to be compared to (potentially) all others and/or already constructed contigs (Pop 2009). Hashing and indexing functions can drastically increase alignment efficiency (Li and Homer 2010), but they cannot eliminate the polynomial nature of growth with respect to the number of input sequences. Eulerian graph-based methods can reduce the time of parsing input data into a graph structure that can (ideally) be efficiently traversed to yield an assembly (Pop 2009). However, the complexity of the resulting graph, and therefore the difficulty of its traversal, rapidly increases given either erroneous data or true genomic polymorphism. While these complicating factors are either infrequent or easily correctable in the case of single genomes, they are intrinsic components of metagenomes, and they therefore encourage the metagenomic investigator to choose between inappropriate data corrections or inefficient whole-dataset assembly.

With respect to the size of the input dataset, the linear disadvantage of PRICE (mapping) was countered by a polynomial advantage (reduced input for each seeded assembly job). A similar advantage could have been gained by pre-sorting data of interest away from irrelevant data. The goal of PRICE was to provide such an algorithmic advantage for assembly even if only a tiny subset of the data of interest could be identified ahead of time. However, the added cost of repeated mapping through a series of assembly steps limited the expected efficiency advantage of PRICE to cases where the initial design assumptions hold: when an investigator is interested in a particular species that comprises a small fraction of a much larger, more complex dataset, and that subset of data cannot be reliably identified ahead of time.

### PRICE as a tool for the *in silico* purification paradigm

The purpose for which PRICE was designed, the discovery of viruses through sequencing of the viral genome from a clinical or other metagenomic sample, is intrinsically hampered by the metagenomic nature of the genetic source material. Traditionally, the targeting of viral genome would occur at the lab bench. Sample-processing techniques such as particle-size filtration or density-based enrichment by centrifugation increase the frequency of viral genomes by depleting other components of the metagenomic mixture but require that the sought virus have the morphology enriched by the technique (Segura *et al.* 2011). Molecular techniques such as virus-specific polymerase chain reaction (PCR) or microarray hybridization capture provide even more powerful molecular enrichment for viral nucleic acid, but they require very specific prior knowledge about the sequence being sought that can hamper the discovery of highly novel pathogens (Tang and Chiu 2010; Mahony *et al.* 2011). *In vitro* viral culture can provide tremendous viral amplification without specific prior molecular knowledge. However, the specific requirements for host cell biology to support viral replication makes the establishment of such a culture system naïvely improbable, as evidenced by longstanding difficulties establishing suitable systems for the study of replication by hepatitis C virus (Jones and Mclauchlan 2010) and human papillomavirus (Schiller *et al.* 2010). In all cases, these techniques reduce the metagenomic complexity of the sample to enable discovery by imposing constraints on the allowed properties of the pathogens to be discovered.

The low cost of modern DNA sequencing at large scale has facilitated a paradigm shift away from physical to *in silico* enrichment techniques. Rather than anticipating the properties of a particle or genome of interest, total metagenomic data can be collected and sorted afterward for properties of interest. *Versus in vitro* methods, *in silico* enrichment offers the advantage of not destroying any information from the original full dataset, allowing it to be repeatedly queried *ad infinitum* for sequences with new properties. Possibly the most important advantage of *in silico* over physical enrichment is its foundation of information content. The genomics era has framed evolution, the defining process of biological systems, in terms of information theory, propagation, retention, and entropic degradation (Lander 2005). Although many of the physical properties of virus particles that are leveraged for physical enrichment are selectively maintained, their utility for enrichment is often arbitrary with respect to the information impacted by natural selection. Worse, pathogens physically protect their best-conserved structures from host immune systems (Ploegh 1998), a property that can also shield them from physical enrichment tools. *In silico*, appropriate attention can be focused on those parts of a genome that are also the focus of purifying selection: for instance, by examination of the translated amino acid sequence of the nucleic acid under scrutiny, or further by focusing on those aligned positions whose function has been widely conserved across the family of proteins with similar functional roles.

The selection of appropriate sequences to seed PRICE assemblies represents a parallel bioinformatic challenge to the targeted assembly implemented by PRICE. The various examples presented in this paper highlight the pros and cons of alternative seed selection strategies. When searching for novel biological entities, the similarity of new sequences to known sequences/motifs can be slight, implying that database searches should be performed with lenient requirements. However, such leniency can also attract false-positive matches that will seed the assembly of undesired contigs. The LSV2 and keratin assemblies were each seeded with individual reads, demonstrating the ability of PRICE to function given only a small amount of initial information. This ability would give the user the option of using highly stringent similarity requirements, the kind of requirements that would identify very few satisfactory sequences. In the case of the larger KSHV genome, several seeds were evenly spaced at wide (5-kb) intervals. Although those seeds provided a clean and complete assembly, the inability of all contigs to join highlighted the reality that there were practical limits to the contig extension that PRICE could accomplish, and the presence of regions with insufficient data for assembly can generate inaccessible genomic islands that would be missed by seeds distributed too sparsely. In addition, the KSHV assembly took many more cycles than the LSV2 or keratin assemblies, highlighting the performance advantage of lenient database searches that lead to higher seed density.

A large advantage of the iterative assembly protocol of PRICE that has not been highlighted was its ability to make use of assembly that has already been performed. All three of the assemblies presented here were seeded with short reads. As has been discussed, it can be difficult to find meaningful similarity between short reads and distantly related reference sequences. Our application of whole-dataset assemblers to the LSV2-containing bee-associated metagenome illustrated the advantages provided by a targeted assembly strategy toward obtaining a full viral genome sequence. However, it also demonstrated the ability of all of the whole-dataset assemblers used to generate fragments of correctly assembled sequence far longer than individual reads. Those contigs, if deemed interesting, could easily have served as seed sequences for PRICE assembly. The utility of naïve whole-dataset assembly can be maximized by placing it in the context of a pipeline, with PRICE at the end to compensate for the limitations of the whole-dataset approach.

The role for PRICE in expanding the paradigm of *in silico* purification/enrichment was to amplify the power of information-based purifications by providing a mechanism for co-purification of genomically associated sequences. The examples presented here, in combination with the published applications to real-world metagenomic problems, highlighted the ability of PRICE to maximize the amount of information that can be gathered about a biological sequence and its (meta)genomic context given identification of a short sequence of potential interest from a large, complex library of sequences. They also highlighted the ability of PRICE to accommodate the challenges that are both intrinsic to such problems (complexity of datasets in terms of diversity of the source material) and that reflect current shortcomings of metagenomic data (unevenness of coverage, even across a single molecular species). PRICE was designed pragmatically, to address these practical problems that are common to contemporary deep-sequence datasets. PRICE shifts burden away from the bench (the generation of a perfect library) and onto the computer (handling problematic data). By doing so, PRICE improves the likelihood that an arbitrary dataset will produce useful data of interest and therefore reduces the time, financial cost, and risk associated with metagenomics projects. Reducing the costs and risks of metagenomic analyses should empower a wider community of scientists to engage in such projects and help democratize the field of metagenomics.

## Supplementary Material

Supporting Information
