# Supplementary material for: PRICE: Software for the Targeted Assembly of Components of (Meta) Genomic Sequence Data
Source: G3 (Bethesda). 2013 May 1;3(5):865–80. doi: 10.1534/g3.113.005967 (PMC3656733; doi:10.1534/g3.113.005967)
Supplement: Supporting Information [file supp_3_5_865__index.html]

PRICE: Software for the Targeted Assembly of Components of (Meta)Genomic Sequence Data — PRICE: Software for the Targeted Assembly of Components of (Meta) Genomic Sequence Data — Supporting Information 

# PRICE: Software for the Targeted Assembly of Components of (Meta) Genomic Sequence Data

## Supporting Information for Ruby, Bellare, and DeRisi, 2013

**Files in this Data Supplement:**

- File S1 - Fasta-formatted text file of seed sequences for the KSHV genome assembly, extracted from the reference genome45 (.fasta, 3 KB)
- File S2 - Fasta-formatted text file of the contigs generated by the KSHV genome assembly (.fasta, 133 KB)
- File S3 - Detailed command-line information for assemblies, including data on computational resource consumption (.txt, 4 KB)
